# Maxillary Sinus Puncture: A Traditional Procedure in Decline—Insights from SHIP

**DOI:** 10.3390/jcm14155578

**Published:** 2025-08-07

**Authors:** Fabian Paperlein, Johanna Klinger-König, Chia-Jung Busch, Christian Scharf, Achim Georg Beule

**Affiliations:** 1 Department of Otorhinolaryngology, Head and Neck Surgery, University Medicine Greifswald, 17475 Greifswald, Germany; 2 Department of Psychiatry and Psychotherapy, University Medicine Greifswald, 17475 Greifswald, Germany; 3 Department of Otorhinolaryngology, Head and Neck Surgery, University Hospital Münster, 48149 Münster, Germany

**Keywords:** maxillary sinus puncture, rhinosinusitis, sinonasal quality of life, SNOT-20-D, historical medical practice, population-based study, SHIP, otorhinolaryngology

## Abstract

**Background:** Maxillary sinus puncture (MSP), once a cornerstone for diagnosing and treating acute rhinosinusitis (ARS), has declined with the rise in less invasive techniques. This study explores MSP trends, its association with age, and long-term effects on quality of life using data from the Study of Health in Pomerania (SHIP). **Methods:** Data from SHIP-START-2 (*n* = 2332), SHIP-START-3 (*n* = 1717), and SHIP-TREND-0 (*n* = 4420) cohorts were analyzed to assess MSP prevalence, demographic correlations, and quality- of-life impacts using SNOT-20-D, EQ-5D-3L, and SF-12. **Results:** MSP prevalence was higher in older SHIP-START cohorts (11.2% in START-2) compared to SHIP-TREND-0 (9.5%), reflecting its historical decline. The procedure was more frequently reported by participants aged > 60 years (e.g., 14.0% in START-2) than by younger groups (<40 years: 3.5% in START-2). MSP was associated with increased SNOT-20-D scores across cohorts (e.g., +0.28 in START-2, *p* < 0.001) and minor reductions in EQ-5D-3L and SF-12 mental health scores, indicating greater symptom burden but limited general health impact. The age- and time-related decline in MSP highlights its diminishing role in modern practice. **Conclusions:** While MSP offers diagnostic insights and serves as an indicator for ARS, its modest impact on long-term quality-of-life underscores the need for alternative, minimally invasive techniques for sinonasal conditions.

## 1. Introduction

Maxillary sinus puncture (MSP) was long considered the gold standard for diagnosing and treating acute rhinosinusitis (ARS), particularly in the late 20th century [1,2]. Historically used both diagnostically and therapeutically through irrigation, MSP has largely been replaced by endoscopic middle meatal cultures (EDMMs), which are less invasive, more accessible, and associated with reduced patient discomfort and risk [1]. While MSP remains in use in some countries undergoing procedural refinement [3], contemporary European and national guidelines now recognize it only as a secondary option, typically for microbiological sampling in more severe ARS cases managed in secondary or tertiary care settings [4,5].

Despite this decline, MSP use may have persisted longer in rural or resource-limited settings, such as those formerly in East Germany, due to historical practice patterns and systemic differences in healthcare infrastructure. We hypothesize that MSP was more prevalent in these regions and age groups with limited access to newer diagnostic techniques, and may serve as a retrospective proxy marker for severe ARS episodes. To explore this hypothesis, we analyzed data from the Study of Health in Pomerania (SHIP), a large-scale, longitudinal, and population-based health study in northeastern Germany. The SHIP was established to investigate the prevalence of chronic diseases, risk factors, and their interactions in the general adult population. The assessments include standardized face-to-face interviews, self-report questionnaires, physical examinations, and various biospecimens. It comprises several cohorts [6,7]:

- **SHIP-START**: The original cohort with baseline data collected between 1997 and 2001, followed by START-2 (2008–2012) and START-3 (2014–2016);
- **SHIP-TREND**: An independent cohort established in 2008 (TREND-0: 2008–2012) to refresh the population sample;
- **SHIP-NEXT**: Launched in 2021 but excluded from this analysis due to ongoing data collection.

Participants were randomly selected from regional registries and underwent standardized clinical assessments, interviews, and validated questionnaires, including the Sinonasal Outcome Test—German version (SNOT-20-D), the EuroQol 5-Dimension 3-Level questionnaire (EQ-5D-3L), and the 12-Item Short Form Survey (SF-12).

### Objective

This study aims to assess age- and time-related trends in MSP use across SHIP cohorts and to evaluate its long-term association with sinonasal symptom burden and quality of life. Our findings offer insights into the historical role of MSP in ARS management and its potential utility as an epidemiological indicator in population health research.

## 2. Materials and Methods

### 2.1. Study Design and Population

The analyses used data from the Study of Health in Pomerania (SHIP), a population- based cohort study conducted in northeastern Germany to examine the prevalence of chronic diseases, risk factors, and healthcare utilization in the general adult population [7]. We included participants from two distinct SHIP cohorts:

- **SHIP-START**, the first cohort, with baseline data collected in 1997–2001 (START-0; *n* = 4308). We used follow-up data from START-2 (2008–2012; *n* = 2333) and START-3 (2014–2016; *n* = 1717);
- **SHIP-TREND-0**, the baseline data of an independent second cohort recruited in the same region (2008–2012; *n* = 4420).

The START and TREND cohorts were drawn from the same geographic area but included non-overlapping participants. Data from START-3 were used only for longitudinal consistency analysis and excluded from cross-sectional analyses to avoid repeated- measurement bias. Ethics approval was obtained for all cohorts and waves, and participants provided written informed consent. Access to the dataset was granted through formal application to the SHIP data committee.

### 2.2. Assessment of Sinonasal History and Interventions

In SHIP-START-2 and START-3, participants completed a 20-item sinus-specific questionnaire designed to capture sinonasal symptoms, structural abnormalities, surgical history, and interventions. For the present study, we selected six items focusing on procedural history—namely, maxillary sinus puncture (MSP), septoplasty, and paranasal sinus surgery—which directly align with our study objectives.

The timing of each reported procedure was recorded via open-ended responses, allowing participants to indicate either the year or age at which the intervention occurred. Other questionnaire items, although relevant to broader rhinologic phenotyping, were excluded to maintain a clear focus on intervention trends and quality-of-life outcomes.

In SHIP-TREND-0, only two of the six procedural questions—those concerning MSP and paranasal sinus surgery—were included, due to financial constraints limiting questionnaire length.

The six selected items were as follows:

1. Have you ever had your nasal septum straightened? (0 = no, 1 = yes);
2. If yes, when? (open-ended response);
3. Have you ever undergone a maxillary sinus puncture?* (0 = no, 1 = yes);
4. If yes, when? (open-ended response);
5. Have you ever had surgery on the paranasal sinuses?* (0 = no, 1 = yes);
6. If yes, when? (open-ended response).

Only the questions marked with an asterisk (*) were included in SHIP-TREND-0. All six items were included in SHIP-START-2 and SHIP-START-3.

### 2.3. Quality-of-Life Instruments

Quality of life was assessed using three validated tools:

- **SNOT-20-D:** This 20-item questionnaire evaluates symptom burden and quality-of-life impact in individuals with sinonasal complaints. It includes items on nasal obstruction, facial pressure, and olfactory function. The German version (SNOT-20-D) is a standardized translation of the original SNOT-20 developed by Piccirillo et al. [8] and was the best-available tool at the time of SHIP-START for sinonasal health assessment in a German-speaking population. Higher scores indicate greater symptom severity; a mean score of ≥0.8 was defined as the minimal clinically important difference (MCID);
- **EQ-5D-3L:** This is a standardized measure of general health across five domains: mobility, self-care, usual activities, pain/discomfort, and anxiety/depression. Index scores were calculated using German-specific value sets [9,10], with higher values indicating better health. The EQ-5D is validated for use in CRS research and general populations [11];
- **SF-12:** This a concise, validated tool for assessing subjective physical and mental health. It produces two summary scores (physical and mental component summaries). Higher scores reflect better perceived health status [12].

### 2.4. Use of AI Tools

OpenAI’s ChatGPT-4 was used to support language refinement and formatting during manuscript preparation. No data analysis or scientific interpretation was performed using AI tools.

### 2.5. Statistical Analysis

All analyses were performed using R version 4.2.1. Descriptive statistics are presented as means (M) with standard deviations (SDs) for continuous variables, and counts (n) with percentages (%) for categorical variables.

Linear regression models were used to evaluate the association between MSP and quality-of-life outcomes (SNOT-20-D, EQ-5D-3L, and SF-12). Models were adjusted for age, sex, smoking status, and allergy history. The main exposure variable was intervention status (MSP vs. no intervention), with the latter used as the reference group.

To explore the effect of the timing of the intervention, we performed additional linear regressions using the following:

- Years since MSP;
- Age at MSP;
- Interaction: years since × age at MSP;
- Interaction: age at MSP × sex.

Timing analyses were restricted to SHIP-START-2, as TREND-0 did not include timing data. For descriptive purposes, we also examined the distribution of interventions by age and calendar year and assessed the consistency of MSP reporting between START-2 and START-3 in overlapping participants.

## 3. Results

### 3.1. Cohort Characteristics and MSP Prevalence

In START-2, 2306 out of 2332 participants provided valid data on MSP use in earlier decades, of whom 258 (11.2%) reported undergoing the procedure. In TREND-0, 4235 out of 4420 participants provided valid data, with 401 (9.5%) reporting MSP. The mean age was higher in START-2 (57.4 years) compared to TREND-0 (52.0 years), consistent with START-2 being a follow-up cohort and TREND-0 being a baseline population. The gender distribution was comparable across cohorts (53.0% women in START-2; 51.5% in TREND-0).

**Figure 1:**
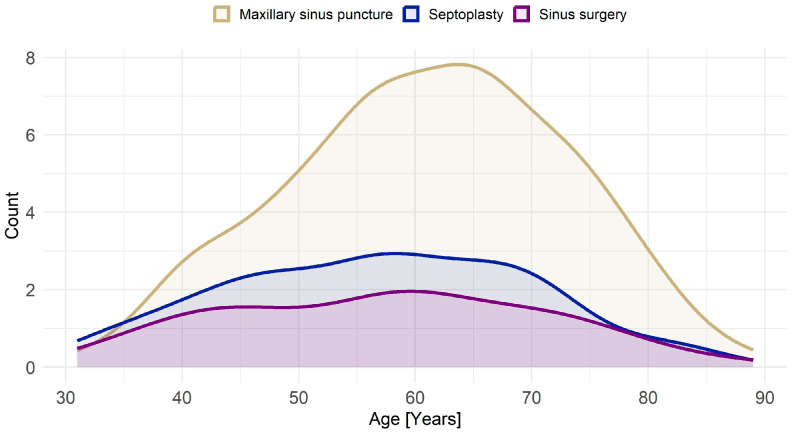
Age distribution of sinonasal procedures. Procedures include MSP (maxillary sinus puncture), STP (septoplasty), and ESS (endoscopic sinus surgery). The graph shows MSP being most frequently performed in older age groups, peaking around 60–70 years, while STP and ESS exhibit a more balanced distribution across ages, with slightly higher prevalence in middle-aged individuals.

Figure 1 visualizes the age-related distribution of sinonasal procedures across both cohorts. MSP shows a clear increase with age, peaking in the >60 group (14.0% in START-2 and 13.8% in TREND-0), while being rare among participants < 40 years (3.5% in START-2, 3.3% in TREND-0). In contrast, septoplasty (STP) and endoscopic sinus surgery (ESS) are more evenly distributed across age groups, with STP slightly more common among younger individuals. These age patterns reflect historical shifts in clinical practice, where MSP was more common in prior decades. Self-reported allergy prevalence was substantially higher in TREND-0 (29.3%) than in START-2 (9.4%). Smoking was also more common in TREND-0 (26.9%) than in START-2 (20.8%), particularly among younger participants.

Quality-of-life measures showed only minor cohort differences. EQ-5D-3L index scores averaged 0.9 in both cohorts. SNOT-20-D mean scores were 0.7, with higher values in women and younger participants. SF-12 general and mental health scores were similar across cohorts, with slightly lower physical scores in older participants. These baseline characteristics are summarized in Table 1.

### 3.2. Longitudinal Consistency and MSP Timing

Longitudinal data from 1596 participants who provided MSP information in both START-2 and START-3 revealed that 181 individuals reported MSP consistently. Of these, 72 provided timing data in both waves, with 26.4% reporting the exact same year and another 26.4% differing by only one year. An additional 33 participants reported MSP in START-3 but not in START-2, and most dated their procedures before 2008, suggesting recall limitations rather than actual new interventions.

**Figure 2:**
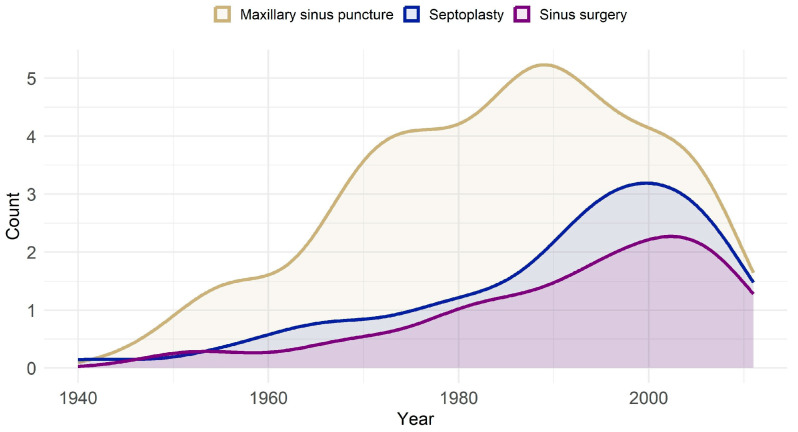
Trends in sinonasal procedures, including MSP, STP, and ESS, from 1940 to 2012. The graph illustrates the peak usage of MSP in the late 20th century, followed by a decline in recent years, alongside more gradual trends in STP and ESS.

The distribution of MSP by year shows a clear historical peak in the mid to late 20th century, with most interventions dated before 2000. This decline is visualized in Figure 2.

As illustrated in Figure 1, cross-sectional age distributions mirror these longitudinal patterns. Older adults, who likely underwent MSP in earlier decades, show the highest prevalence, reinforcing the age–procedure relationship.

### 3.3. Impact of MSP on Quality of Life

MSP was consistently associated with a greater sinonasal symptom burden. In START- 2, MSP participants had a mean SNOT-20-D score 0.28 points higher than non-MSP participants (*p* < 0.001); in TREND-0, the difference was 0.17 points (*p* < 0.001). Moreover, MSP participants were significantly more likely to meet or exceed the minimal clinically important difference (MCID) threshold of *≥* 0.8, with odds ratios of 2.41 (95% CI: 1.62–3.57) in START-2 and 1.56 (95% CI: 1.18–2.07) in TREND-0 (Table 2).

General health measures were less affected. EQ-5D-3L scores were marginally lower in START-2 MSP participants (*β* = −0.02, *p* = 0.042), while SF-12 mental health scores also declined slightly (*β* = −1.24, *p* = 0.039). No significant associations were observed for general or physical health scores in TREND-0.

**Figure 3:**
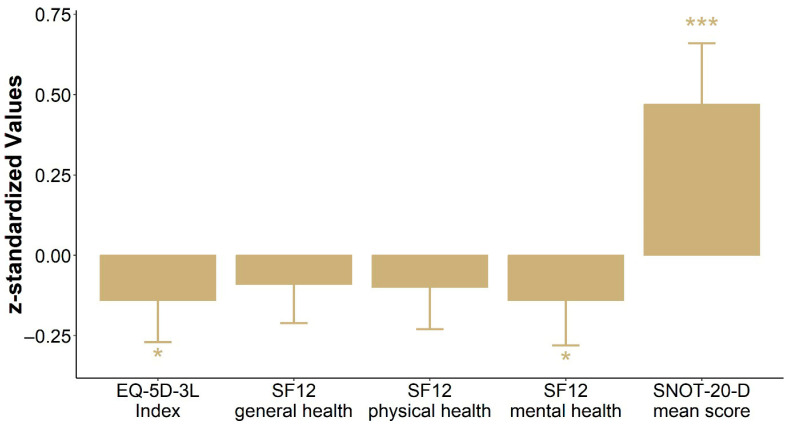
Impact of maxillary sinus puncture (MSP) on quality of life: comparison across multiple domains. Results are presented as standardized coefficients (z-scores) for the EQ-5D-3L index, SF-12 (general health, physical health, and mental health), and SNOT-20-D mean score to enable direct comparability between the scores. For the EQ-5D-3L and SF-12, positive values indicate better quality of life/subjective health, whereas for the SNOT-20-D, negative values indicate fewer symptoms. Changes are presented relative to no intervention. Significant differences are denoted by * *p <* 0.05 and *** *p <* 0.001.

The combined impact of MSP across different health domains is visualized in Figure 3. Here, standardized regression coefficients (z-scores) allow for comparison across scales. SNOT-20-D scores show the strongest deviation, clearly indicating increased symptom burden in MSP participants. Effects on general health and mental health were more modest, though directionally consistent.

Further exploratory analyses in START-2 assessed whether age at intervention or time since MSP affected outcomes. Table 3 shows a borderline association between age at intervention and EQ-5D-3L scores (*p* = 0.053), but no robust patterns across other measures.

## 4. Discussion

### 4.1. Significance of the Study

This study is one of the first to investigate maxillary sinus puncture (MSP) using a large, population-based epidemiological dataset with longitudinal data. Our analysis of over 6700 participants across two major German cohorts (SHIP-START-2 and SHIP-TREND-0) reveals both historical and age-related trends in MSP use, and provides rare insights into its long-term association with sinonasal and general quality of life.

We demonstrate that MSP, although now rarely used and largely reserved for selected or recalcitrant cases, may serve as a proxy marker for prior episodes of severe acute rhinosinusitis (ARS). Our findings suggest that MSP is associated with a small but consistent long-term reduction in sinonasal quality of life, even decades after the procedure.

### 4.2. Historical Trends and Age Distribution

**Figure 4:**
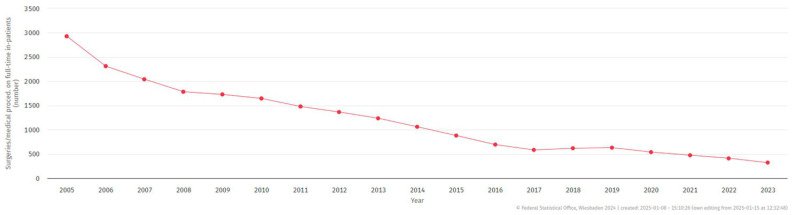
Trend in maxillary sinus puncture (MSP) performed on inpatients in Germany (2005–2023). The data, categorized by surgical and procedural codes (1–4-digit hierarchy), were sourced from case-based hospital statistics (DRG). © Federal Statistical Office of Germany (Destatis), 2024. Status: 8 January 2025, 15:10:26.

A central finding of this study is the temporal decline in MSP, both in national inpatient data and within our population sample. Figure 2 and Figure 4 show a sharp reduction in MSP procedures in Germany—from 2927 in 2005 to just 321 in 2023. This trend reflects updates in clinical guidelines and a broader shift toward minimally invasive alternatives such as endoscopic sinus surgery (ESS) and endoscopically directed middle meatal cultures (EDMMs) [4,5,13,14,15].

Despite its decline, MSP remains in use in specific clinical contexts, including intensive care units [16], for young children under general anesthesia [17], and in some Eastern European countries where refinement continues [3]. Globally, MSP has been largely replaced by less invasive techniques such as endoscopic sinus surgery, and published data on its trends remain limited. Neto et al. [16] observed that while MSP was historically important for managing hospital-acquired rhinosinusitis, its role has diminished as newer approaches became available. In Brazil, national health data (DATASUS) from 2008 to 2025 recorded no procedures under its specific code, likely reflecting replacement by endonasal interventions or reclassification under broader sinonasal codes. These contrasting patterns underscore MSP’s role as a marker of changing medical practice, shaped by guidelines, infrastructure, and training.

As shown in Figure 1, MSP was disproportionately more common in older adults, particularly those aged 60 years and above. This reflects its widespread use in earlier decades [18], prior to the adoption of ESS as standard practice. In contrast, procedures like septoplasty (STP) and sinus surgery (SS) show a more balanced age distribution.

### 4.3. Reliability of Self-Reported MSP and Recall Bias

Longitudinal data from participants who completed both START-2 and START-3 suggest a moderate level of recall accuracy. Among those reporting MSP at both time points, approximately 40% recalled the same or nearly the same year of intervention. This level of consistency is notable given the average time lag of 26 years since the procedure.

Reporting discrepancies—seen at a consistency rate of 52.78%—highlight typical challenges in long-term recall. Factors such as the perceived importance of MSP, its outpatient nature, and cognitive decline with age may influence accuracy [19]. The inability to cross-validate responses with medical records due to German privacy law further limits validation, although this is a common challenge in population-based research.

### 4.4. Impact on Quality of Life

Our findings show a statistically significant association between MSP and reduced sinonasal quality of life, measured by SNOT-20-D. Participants with a history of MSP reported higher symptom scores and were more likely to exceed the clinically important difference threshold (see Table 2).

This association persisted across both cohorts and was evident years after the procedure, indicating a long-term burden. Although general health measures (EQ-5D-3L, SF-12) showed more modest changes, slight reductions in physical and mental health scores were observed in MSP groups (see Figure 3).

Given MSP being primarily used for severe ARS, these results suggest that ARS may have lasting effects, possibly contributing to or overlapping with chronic rhinosinusitis (CRS). Our findings align with previous studies reporting long-term quality-of-life burdens from sinonasal disease [20,21], but extend this knowledge by showing such effects long after intervention.

These findings support the hypothesis that severe ARS—often treated with MSP—may contribute to or accelerate CRS development, a pathway not well characterized in epidemiologic studies [5].

Similarly, general quality of life showed a limited but consistent decline in MSP patients across EQ-5D-3L and SF-12 domains. While most QoL studies measure short-term outcomes (e.g., within 2 weeks post-intervention), our long-term design offers unique value. For instance, Stjärne et al. [22] reported residual EQ-5D burden in ARS patients weeks after onset, and Sathananthar et al. [23] observed symptom relief 20 months after canine fossa puncture (CFP), especially in cases of severe maxillary sinus disease.

QoL tools such as SNOT-16 and EQ-5D have shown acceptable sensitivity but were not developed for long-term ARS tracking [24,25]. Al-Asadi et al. [26] further showed elevated SNOT-22 in recurrent ARS cases. Our findings, using SNOT-20-D, expand this evidence base by linking MSP to persistent symptom burden in a population setting.

This supports MSP as a retrospective marker of past severe ARS and suggests a potential link to CRS evolution, meriting further longitudinal study.

### 4.5. Confounding Variables

In our cohorts, allergy prevalence increased over time—from 9.4% in START-2 to 29.3% in TREND-0—which may reflect both a true rise in incidence and improved diagnostic awareness, consistent with global trends [27]. Smoking rates also differed by age and cohort, with younger participants in TREND-0 reporting a higher smoking prevalence (26.9%) compared to START-2 (20.8%). These patterns align with broader public health data: in 1995, smoking was most prevalent among individuals aged 30–49 [28], while recent CDC data from 2022 show the highest cigarette use among adults aged 45–64, and e-cigarette use was concentrated among those aged 18–24 [29].

Given the known associations between allergies, smoking, and sinonasal symptom burden, we adjusted for both variables in all regression models. While they may contribute to the baseline variation in quality-of-life scores, their inclusion as covariates reduces the likelihood of residual confounding.

### 4.6. Limitations

This study has several limitations: First, recall bias may have affected self-reported procedural data, though the relatively high agreement between START-2 and START-3 lends support to the reliability of these reports. Second, selection bias may have occurred if patients recently operated on were not yet captured in follow-up rounds. Third, the lack of validation with clinical records is a recognized constraint in German cohort studies due to decentralized health data systems. Additionally, MSP cases were relatively few compared to the total sample, which limits the power to detect small subgroup effects. The dataset also lacked details on sinus surgery types, which likely ranged from minimally invasive techniques (e.g., FESS) to more extensive procedures performed in earlier decades. Finally, quality-of-life instruments like SNOT-20-D were originally validated for CRS, not ARS, though they remain widely used due to the lack of ARS-specific long-term tools.

## 5. Conclusions

This study confirms that MSP—a declining but historically important procedure—remains detectable in population surveys and correlates with reduced sinonasal quality of life years later. Approximately 40% of participants accurately recalled the procedure timing decades after the event, offering a promising benchmark for memory-dependent epidemiologic work.

As one of the first large-scale studies to examine long-term quality-of-life effects following MSP, our results support its use as a retrospective proxy for severe ARS. These findings underline the need for more longitudinal studies to understand how acute infections may evolve into chronic conditions and affect general health outcomes over time.

## Figures and Tables

**Table 1:** Descriptive statistics for START-2 and TREND-0 cohorts by demographic groups.

START-2							
Variable	N	Overall	Men	Women	<40 Years	40–60 Years	>60 Years
Sex [women]	2332	1235 (53.0)	–	–	148 (57.4)	581 (54.4)	506 (50.3)
Age [years]	2332	57.4 (13.7)	58.3 (13.8)	56.5 (13.5)	35.8 (2.5)	50.2 (6.1)	70.5 (6.8)
Allergies [yes]	2309	217 (9.4)	73 (6.7)	144 (11.8)	24 (9.3)	105 (9.9)	88 (8.9)
Current smoking [yes]	2323	484 (20.8)	232 (21.2)	252 (20.5)	81 (31.4)	309 (29.0)	94 (9.4)
EQ-5D-3L index	2301	0.9 (0.2)	0.9 (0.1)	0.9 (0.2)	0.9 (0.1)	0.9 (0.1)	0.9 (0.2)
SF12 general health	2318	3.0 (0.7)	3.0 (0.7)	3.0 (0.7)	3.4 (0.7)	3.1 (0.7)	2.9 (0.6)
SF12 physical health	2262	47.0 (9.1)	46.9 (9.3)	47.1 (8.9)	50.7 (7.0)	48.5 (8.3)	44.4 (9.7)
SF12 mental health	2262	52.5 (8.7)	54.0 (7.7)	51.2 (9.3)	51.7 (8.6)	51.9 (8.7)	53.4 (8.6)
SNOT-20-D summary score	1247	13.1 (12.2)	11.0 (10.5)	15.1 (13.4)	15.1 (12.8)	14.0 (12.8)	11.2 (10.8)
SNOT-20-D mean score	1247	0.7 (0.6)	0.6 (0.5)	0.8 (0.7)	0.8 (0.6)	0.7 (0.6)	0.6 (0.5)
SNOT-20-D MCID [yes]	1247	407 (32.6)	161 (26.3)	246 (38.7)	66 (43.7)	226 (34.9)	115 (25.7)
Maxillary sinus puncture [yes]	2306	258 (11.2)	116 (10.7)	142 (11.6)	9 (3.5)	111 (10.5)	138 (14.0)
Age at puncture [years]	218	35.7 (15.8)	37.7 (17.9)	34.2 (13.6)	26.8 (5.4)	30.7 (12.1)	40.2 (17.3)
Time since puncture [years]	218	25.1 (15.2)	25.7 (16.7)	24.6 (14.0)	9.1 (5.2)	20.7 (12.5)	29.7 (15.9)
**TREND-0**							
**Variable**	**N**	**Overall**	**Men**	**Women**	**< 40 years**	**40–60 years**	**> 60 years**
Sex [women]	4420	2275 (51.5)	–	–	582 (53.8)	990 (52.1)	703 (48.9)
Age [years]	4420	52.0 (15.5)	52.6 (15.7)	51.3 (15.2)	31.4 (5.1)	50.3 (6.1)	69.6 (5.7)
Allergies [yes]	4248	1245 (29.3)	443 (21.4)	802 (36.8)	372 (34.8)	557 (29.8)	316 (24.1)
Current smoking [yes]	4398	1183 (26.9)	624 (29.3)	559 (24.7)	458 (42.5)	593 (31.2)	132 (9.3)
EQ-5D-3L index	4369	0.9 (0.1)	0.9 (0.1)	0.9 (0.2)	0.9 (0.1)	0.9 (0.1)	0.9 (0.2)
SF12 general health	4404	3.1 (0.7)	3.1 (0.7)	3.0 (0.7)	3.4 (0.7)	3.1 (0.7)	2.8 (0.6)
SF12 physical health	4310	47.3 (8.8)	47.6 (8.7)	47.0 (9.0)	51.4 (6.1)	47.6 (8.6)	43.7 (9.4)
SF12 mental health	4310	52.4 (8.6)	53.8 (7.9)	51.2 (9.1)	51.5 (7.8)	52.0 (9.0)	53.8 (8.6)
SNOT-20-D summary score	2422	13.8 (12.0)	12.3 (11.3)	15.4 (12.4)	14.2 (12.0)	14.3 (12.5)	12.8 (10.9)
SNOT-20-D mean score	2422	0.7 (0.6)	0.6 (0.6)	0.8 (0.6)	0.7 (0.6)	0.7 (0.6)	0.6 (0.5)
SNOT-20-D MCID [yes]	2422	853 (35.2)	359 (29.5)	494 (41.0)	220 (36.7)	422 (36.0)	211 (32.5)
Maxillary sinus puncture [yes]	4235	401 (9.5)	182 (8.8)	219 (10.1)	35 (3.3)	186 (10.0)	180 (13.8)

**Table 2:** Associations between maxillary sinus puncture (MSP) and quality-of-life outcomes, adjusted for age, sex, smoking status, and allergy history. Significant associations (*p <* 0.05) are highlighted in **bold**. Continuous outcomes are shown as regression coefficients (*β*) with 95% confidence intervals (CIs); binary outcomes are shown as odds ratios (ORs). The reference group comprises participants without MSP.

Outcome	Cohort	N (No MSP)	N (MSP)	Estimate [95% CI]	*p*-Value
EQ-5D-3L index (*β*)	START-2	1919	238	**−0.02 [−0.04; −0.001]**	**0.042**
	TREND-0	3692	397	0.01 [−0.01; 0.02]	0.324
SF-12 general health (*β*)	START-2	1932	241	−0.06 [−0.14; 0.03]	0.193
	TREND-0	3721	400	−0.05 [−0.11; 0.02]	0.178
SF-12 physical health (*β*)	START-2	1883	237	−0.94 [−2.10; 0.22]	0.112
	TREND-0	3650	392	−0.81 [−1.66; 0.04]	0.060
SF-12 mental health (*β*)	START-2	1883	237	**−1.24 [−2.41; −0.07]**	**0.039**
	TREND-0	3650	392	−0.36 [−1.25; 0.52]	0.423
SNOT-20-D mean score (*β*)	START-2	1051	119	**0.28 [0.17; 0.39]**	**8.9 × 10^−^** **^7^**
	TREND-0	2117	231	**0.17 [0.09; 0.25]**	**5.6 × 10^−^** **^5^**
SNOT-20-D MCID (OR)	START-2	1051	119	**2.41 [1.62; 3.57]**	**1.3 × 10^−^** **^5^**
	TREND-0	2117	231	**1.56 [1.18; 2.07]**	**5.4 × 10^−^** **^6^**

**Table 3:** Impact of demographics and time since intervention on quality-of-life measures of START-2: Results are stratified by intervention (maxillary sinus puncture), with F-values and *p*-values indicating the statistical significance of the associations.

Outcome	Statistic	Time Since Intervention	Age at Intervention	Time × Age Interaction	Age × Sex Interaction
	N	216	216	216	216
EQ-5D-3L index	F-value	2.81	2.60	0.20	1.61
	*p*-value	0.041	0.053	0.994	0.188
	N	218	218	218	218
SF-12 general health	F-value	0.80	2.10	0.64	0.90
	*p*-value	0.497	0.101	0.766	0.444
	N	213	213	213	213
SF-12 physical health	F-value	1.24	1.51	1.18	3.05
	*p*-value	0.296	0.214	0.309	0.030
	N	213	213	213	213
SF-12 mental health	F-value	0.92	1.52	1.05	0.24
	*p*-value	0.432	0.209	0.403	0.869
	N	106	106	106	106
SNOT-20-D summary score	F-value	0.64	1.43	0.66	0.63
	*p*-value	0.593	0.239	0.740	0.594

## Data Availability

The data used in this study are part of the SHIP project and are available upon reasonable request from the SHIP data access committee.

## Abbreviations

The following abbreviations are used in this manuscript:

MSP	Maxillary Sinus Puncture
ARS	Acute Rhinosinusitis
CRS	Chronic Rhinosinusitis
SNOT-20-D	Sinonasal Outcome Test (20-item, German version)
EQ-5D-3L	EuroQol 5-Dimension, 3-Level
SF-12	12-Item Short Form Health Survey
MCID	Minimal Clinically Important Difference
SHIP	Study of Health in Pomerania
ESS	Endoscopic Sinus Surgery
EDMM	Endoscopically Directed Middle Meatal Culture
QoL	Quality of Life
CI	Confidence Interval
OR	Odds Ratio
DFG	Deutsche Forschungsgemeinschaft
BMBF	Federal Ministry of Education and Research (Germany)
